# Treatment of immunotherapy-associated pruritus and prurigo nodularis refractory to dupilumab with nemolizumab

**DOI:** 10.1016/j.jdcr.2026.07.020

**Published:** 2026-07-18

**Authors:** Angela J. Luo, Fateme Rajabi, Sara Kashaf, Jonathan S. Leventhal

**Affiliations:** a Department of Dermatology, Yale School of Medicine, New Haven, Connecticut; b Department of Dermatology, Mass General Brigham, Boston, Massachusetts

**Keywords:** IL-31, immune checkpoint inhibitor, prurigo nodularis, pruritus

## Introduction

Pruritus is a common cutaneous adverse event of immune checkpoint inhibitors (ICIs), affecting approximately 30% of patients.^1^ Pruritus occurs in approximately 24% of patients treated with single-agent programmed cell death protein 1 inhibitors and can significantly impair quality of life.^1^ Most cases occur within 3 to 10 weeks of treatment initiation and may present with or without associated primary or secondary skin changes.^1^ For severe cases recalcitrant to conservative therapy with topical steroids and antihistamines, steroid-sparing agents recommended by the National Comprehensive Cancer Network include dupilumab and omalizumab (ICI-Related Toxicities, v1.2026). Recently, a single case report showed that nemolizumab may be effective for ICI-associated pruritus.^1^

Although direct comparative data are lacking, indirect evidence suggests that nemolizumab may provide greater antipruritic effects in prurigo nodularis (PN), whereas dupilumab may be more effective in eczematous phenotypes.^2^^,^^3^ However, data guiding treatment selection in ICI-associated pruritus remain limited.

## Case report

Here, we describe a case that highlights this therapeutic distinction in the context of ICI-associated pruritus. We present a 71-year-old man with recurrent stage IVA large cell neuroendocrine carcinoma of the lung who developed severe pruritus progressing to PN during treatment with carboplatin, etoposide, and atezolizumab. Approximately 7 weeks after treatment initiation, he developed generalized pruritus rated 9/10. Despite 2 months of treatment with clobetasol 0.05% cream twice daily and oral antihistamines, hydroxyzine, and doxepin, symptoms failed to improve. Atezolizumab was held by oncology for debilitating pruritus and rash after a total of 3 months of treatment.

**Fig 1:**
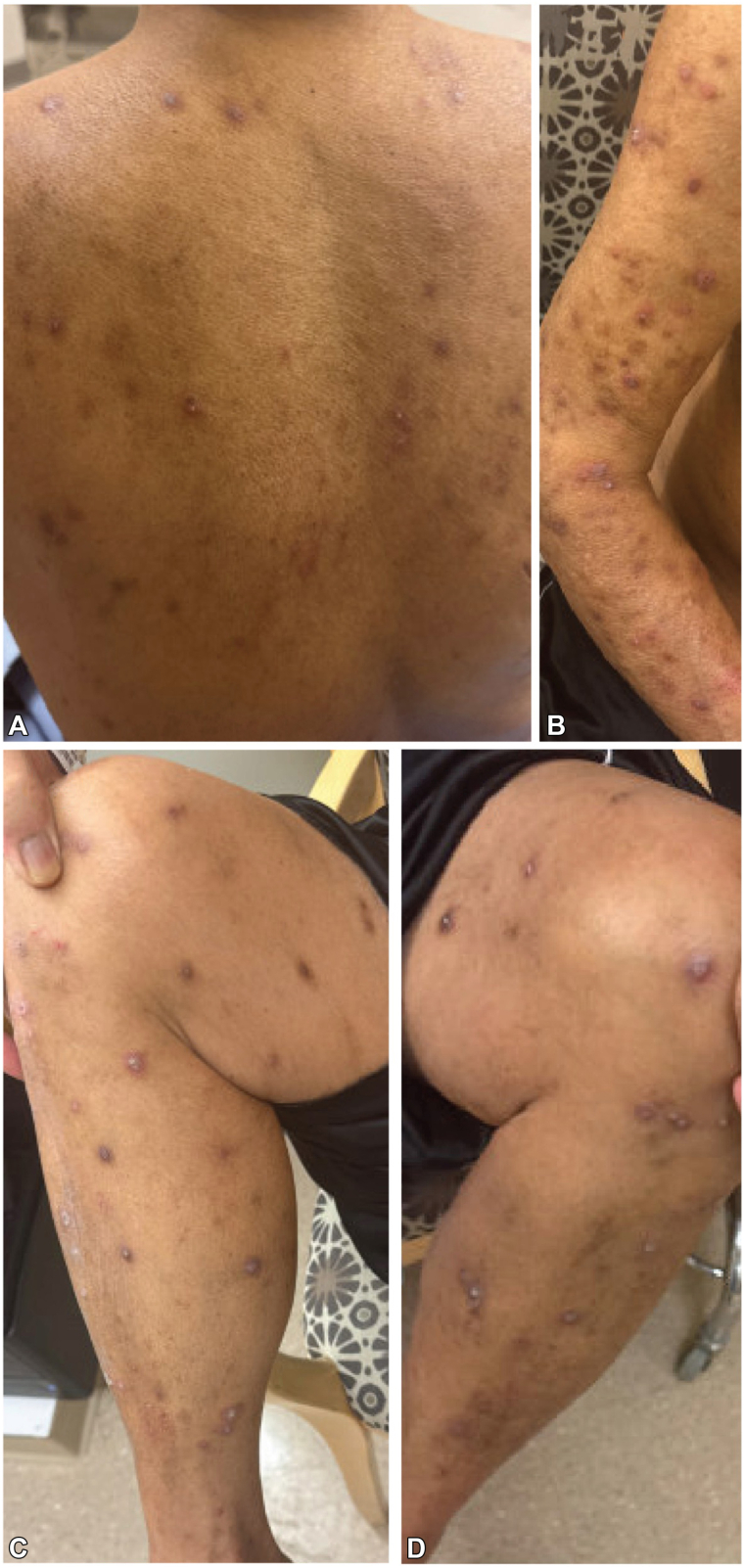
Numerous excoriated papules and nodules with central erosion and crust symmetrically involving the trunk and bilateral upper and lower extremities, consistent with prurigo nodularis in the setting of chronic scratching (**a-d**).

One week later, the patient was evaluated by dermatology. Examination demonstrated numerous pink eroded papules and nodules on the trunk and extensor extremities consistent with PN (Fig 1). BP180/230 titers were normal, arguing against evolving bullous pemphigoid. Other relevant laboratory tests included normal thyroid-stimulating hormone, negative HIV testing, normal complete blood count, and poorly controlled diabetes mellitus (A1C, 8.8%). At 1-month follow-up, pruritus worsened with increased lesion burden, and dupilumab was initiated.

After 3 months of dupilumab therapy, the patient experienced partial improvement in pruritus with persistent PN. Due to progression of his underlying malignancy, systemic cancer therapy was transitioned to tarlatamab, and atezolizumab was not reintroduced.

Despite escalation of dupilumab to weekly dosing, the patient continued to experience nocturnal pruritus and active PN. After 7 months of dupilumab, nemolizumab was initiated, with a 60-mg subcutaneous loading dose (1.0 mg/kg), followed by 30 mg subcutaneously every 4 weeks (0.5 mg/kg) based on his weight of 64 kg.

**Fig 2:**
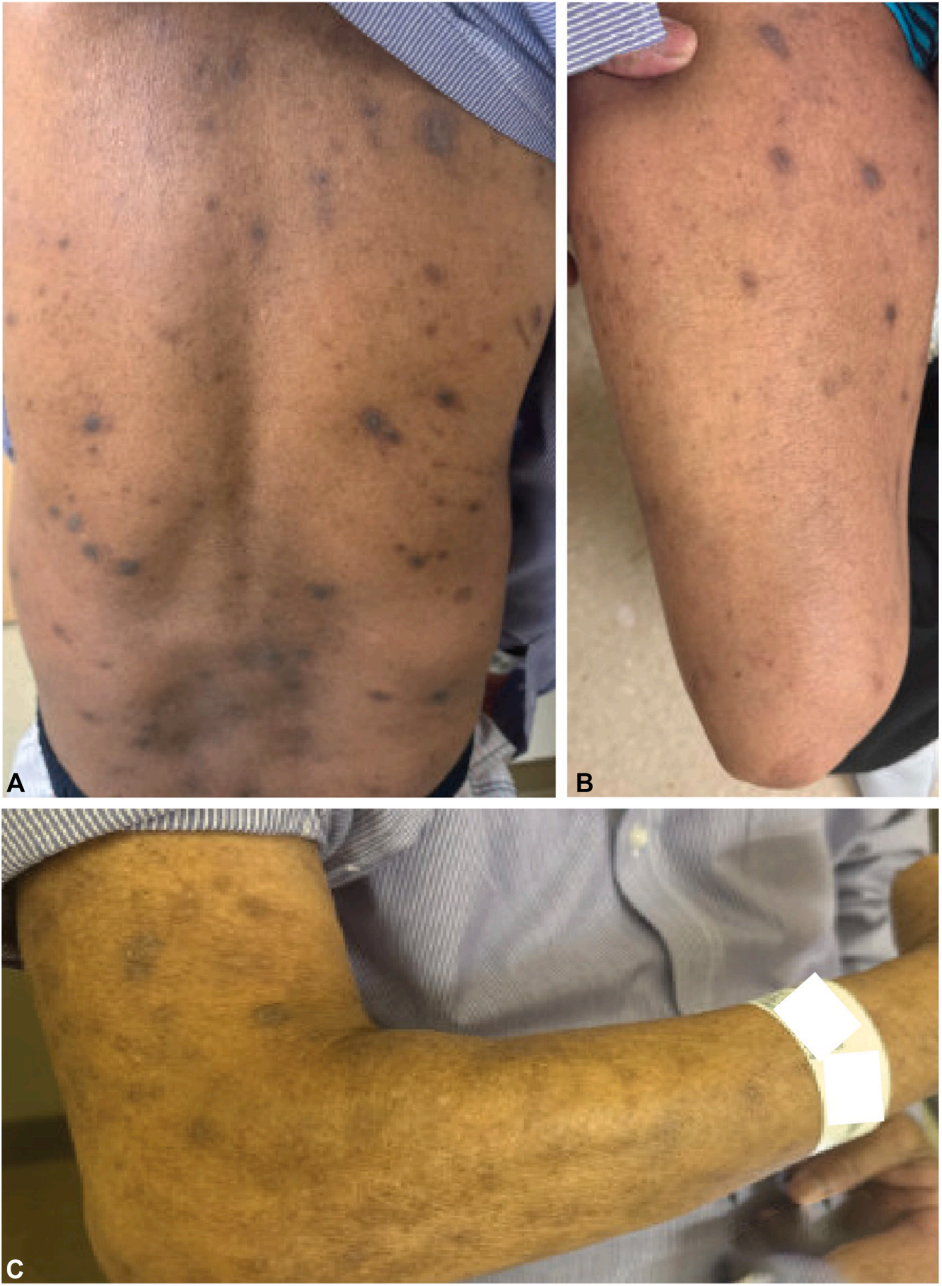
Resolution of prurigo nodularis with flattening of the papules and nodules, leaving residual postinflammatory hyperpigmentation. No erythema, crusting, or erosion (**a-c**).

Within 10 days of starting nemolizumab, the patient reported significant improvement in pruritus, including complete resolution of nocturnal awakenings, with a significant reduction in lesion burden. Examination at 10 days postnemolizumab initiation revealed flattening of previously noted eroded papules and nodules on the trunk and extremities, leaving residual postinflammatory hyperpigmentation (Fig 2). Maintenance dosing at 0.5 mg/kg every 4 weeks was continued, with sustained clinical improvement noted at subsequent follow-up. Concomitant therapies, including topical corticosteroids, tacrolimus ointment, and oral antihistamines, were reduced from scheduled to as-needed use. At the most recent follow-up, approximately 12 months after initial dermatologic evaluation, the patient continued to demonstrate sustained improvement while receiving nemolizumab and had received approximately 10 months of tarlatamab therapy with stable disease on imaging.

## Discussion

The patient’s rapid improvement after initiation of nemolizumab suggests a role for interleukin (IL) 31 signaling in his immunotherapy-associated pruritus. IL-31 is implicated in chronic pruritic disorders and may be relevant in ICI-associated pruritus.^4^ Checkpoint blockade may contribute to immune dysregulation in the skin, and immunologic profiling studies have demonstrated increased IL-4 and IL-31 in eczematous and urticarial phenotypes of ICI-associated pruritus.^4^ The patient’s clinical response to IL-31 receptor blockade suggests that IL-31 signaling may have contributed to his itch.

PN is a complex neuroimmune disorder involving IL-4, IL-13, and IL-31 signaling pathways.^5^ The incomplete response to dupilumab and the marked improvement observed with IL-31 blockade in our patient are biologically plausible, as IL-31 directly activates and sensitizes peripheral sensory neurons.^1^ Preclinical studies suggest that IL-31-driven itch can persist despite IL-4/IL-13 inhibition, indicating that IL-31 may function as an independent itch driver.^6^

Although phase II and III trials of nemolizumab in PN demonstrated favorable efficacy and safety profiles, patients with active malignancy, including those receiving contemporary immunotherapies, were excluded.^7^ Targeted biologics such as dupilumab are increasingly used to manage cutaneous immune-related adverse events in patients receiving immunotherapy, with documented efficacy and safety.^8^ Data regarding concomitant use with tarlatamab remain limited, and additional studies are needed to define the safety of IL-31 blockade in oncologic populations.

Larger prospective studies are needed to define the role of nemolizumab in dupilumab-refractory ICI-associated PN and to guide phenotype-driven treatment selection. The temporal relationship between atezolizumab initiation and onset of pruritus after 7 weeks was consistent with the reported timing of ICI-associated pruritus.^1^ Immunotherapy-associated pruritus may persist beyond treatment discontinuation, reflecting the chronic nature of cutaneous immune-related adverse events.^9^ Although paraneoplastic pruritus was considered given the patient’s underlying neuroendocrine carcinoma, symptoms first developed while imaging demonstrated stable disease following treatment, making a purely paraneoplastic etiology less likely but not excluded. An additional limitation is the absence of histopathologic confirmation. Although the diagnosis of PN is made clinically, reactive perforating collagenosis (RPC) was not formally excluded, as the patient possesses risk factors associated with acquired RPC, including poorly controlled diabetes mellitus and chronic scratching, although no history of chronic kidney disease or dialysis.^10^ Poorly controlled diabetes mellitus may also have contributed to the patient’s pruritus. Consequently, RPC or overlap between RPC and PN cannot be excluded without histopathologic confirmation, and the contribution of diabetes mellitus to the patient’s pruritus cannot be excluded. Another limitation is the absence of serial validated outcome measures. Treatment response was assessed using clinical documentation, patient-reported symptoms, and serial photographs. Because concomitant therapies were available during nemolizumab initiation, clinical improvement cannot be attributed exclusively to nemolizumab. However, the patient reported minimal use of these therapies, which were reduced to as needed after improvement.

Nevertheless, this case highlights the potential efficacy and safety of IL-31 inhibition in severe, recalcitrant immunotherapy-associated pruritus refractory to IL-4/IL-13 inhibition. This case adds to the limited literature supporting IL-31 receptor antagonism for severe immunotherapy-associated pruritus and PN.

## Conflicts of interest

Dr Leventhal has received research funding from Azitra, Inc and OnQuality and has served on advisory boards for Sanofi, Regeneron, and La Roche-Posay. All other authors have no conflicts to disclose.
